# Assessment of antioxidant properties of membrane ultrafiltration peptides from mungbean meal protein hydrolysates

**DOI:** 10.7717/peerj.5337

**Published:** 2018-07-27

**Authors:** Chanikan Sonklin, Natta Laohakunjit, Orapin Kerdchoechuen

**Affiliations:** Division of Biochemical Technology/ School of Bioresources and Technology, King Mongkut’s Institute of Technology Thonburi, Bangkok, Thailand

**Keywords:** Antioxidant peptides, Antioxidant activities, Bromelain, Membrane ultrafiltration, Mungbean meal protein hydrolysate

## Abstract

**Background:**

Bioactive peptides can prevent damage associated with oxidative stress in humans when consumed regularly. Recently, peptides have attracted immense interest because of their beneficial functional properties, safety and little or no side effects when used at high concentration. Most antioxidant peptides are small in size, less than 1 kDa, and contains a high proportion of hydrophobic amino acid. Particularly, tyrosine, leucine, alanine, isoleucine, valine, lysine, phenyalanine, cysteine, methionine and histidine in peptide chain exhibited high antioxidant activity. Mungbean meal protein (MMP) is highly abundant in hydrophobic amino acids. It indicated that MMP might be a good source of antioxidants. Therefore, the objectives were to optimize the conditions used to generate mungbean meal protein hydrolysate (MMPH) with antioxidant activity from bromelain and to investigate the antioxidant activities of different molecular weight (MW) peptide fraction.

**Methods:**

Response Surface Methodology (RSM) was used for screening of the optimal conditions to produce MMPH. After that MMPH was fractionated using ultrafiltration membranes with different MW distributions. Crude-MMPH and four fractions were investigated for five antioxidant activities: 2,2,1-diphenyl-1-picrylhydrazyl (DPPH), hydroxyl, superoxide, ferric reducing antioxidant power (FRAP) and metal ion chelation activity.

**Results:**

The optimal condition to produce the MMPH was 15% (w/w) of bromelain and hydrolysis time for 12 h which showed the greatest DPPH and ABTS radical scavenging activity. After mungbean protein from optimal condition was separated based on different molecular weight, the DPPH radical scavenging activity was the highest for the F4 (less than 1 kDa) peptide fraction. Metal ion chelating activity was generally weak, except for the F4 that had a value of 43.94% at a protein concentration of 5 mg/mL. The F4 also exhibited high hydroxyl and superoxide activities (54 and 65.1%), but moderate activity for ferric reducing antioxidant power (0.102 mmole Fe2+/g protein) compared to other peptide fractions and crude-MMPH. Molecular weight and amino acid were the main factors that determined the antioxidant activities of these peptide fractions. Results indicated that F4 had strong antioxidant potentials.

**Discussion:**

The lowest MW fraction (less than 1 kDa) contributed to the highest DPPH, superoxide, hydroxyl and metal chelation activity because influence of low MW and high content of hydrophobic amino acid in peptide chain. Results from this study indicated that MMPH peptides donate protons to free radicals because they had significantly high DPPH value compared to superoxide, hydroxyl and FRAP, which reactions were electron donation. Moreover, MMPH peptides had the ability to inhibit transition metal ions because of highly abundant glutamic acid and aspartic acid in peptide chain.

## Introduction

Bioactive peptides are usually obtained by enzymatic hydrolysis of food proteins. Normally, peptides within their parent protein chain cannot exhibit bioactive function but these functions can be activated after cleavage by (Chi et al., 2014). Apart from enzymatic hydrolysis, these peptides can also be cleaved from parent proteins by microbial enzymes and during food processing (Pownall, Udenigwe & Aluko, 2010). Enzymatic hydrolysis is a suitable way to produce bioactive peptides without losing nutritional value (He et al., 2013). Additionally, the use of enzymatic hydrolysis of protein can help to control the end product, thereby producing desirable target peptides (Adler-Nissen, 1986). It is an inexpensive method for cleaving proteins to peptides and free amino acids and, the solubility of the peptide products is usually higher than that of the parent protein with their amino acid profile can remain essentially unchanged or probably enhanced in some fractions (Cumby et al., 2008).

An antioxidant is a substance that counteracts the oxidative reaction caused by free radicals (Lobo et al., 2010). The most widely used synthetic antioxidants are butylated hydroxyanisole (BHA), butylated hydroxytoluene (BHT) and *tert*-butylhydroquinone (TBHQ) but the use of synthetic antioxidants is under strict regulation because they are toxic to human health (Ng, Tan & Khor, 2017). In recent years, natural antioxidants have attracted immense interest because of their beneficial functional properties and safety. Natural antioxidants have little or no side effects when used at high concentration (Pownall, Udenigwe & Aluko, 2010; Li et al., 2008). Bioactive peptides are used in the formulation of functional food that can prevent damage associated with oxidative stress in the human body when consumed regularly (Alashi et al., 2014). Recently, many studies are interested in generating bioactive peptides from plant protein sources (Udenigwe & Aluko, 2011). Some factors that influence the bioactivity of peptide are size, amino acid composition and sequence (Arise et al., 2016). Generally, bioactive peptides have two to twenty amino acid residues per chain and often contain high proportion of hydrophobic amino acid (Udenigwe & Aluko, 2011). Almost all bioactive peptide that contain Tyr, Leu, Ala, Ile, Val, Lys, Phe, Cys, Met and His exhibited high antioxidant activity (Wattanasiritham et al., 2016). However, the antioxidant properties of hydrolysates depend on the protease specificity, the degree of hydrolysis (DH), and the nature of the released peptides (molecular weight (MW) and amino acid composition) (Arise et al., 2016).

Mungbean meal is a by-product of vermicelli or green bean noodle industry and has a protein content of 70% (w/w). Owing to its high protein and hydrophobic amino acid contents and abundance, investigating its bioactivity is an important aspect of adding value to this by-product obtained from this growing industry (Sonklin, Laohakunjit & Kerdchoechuen, 2011). Bioactive peptides from mungbean protein were hydrolysed using Alcalase, Neutrase and *Virgibacillus* sp., which the peptides derived from these enzymes, possessed antioxidant and angiotensin I converting enzyme (ACE) inhibitory activities (Lapsongphon & Yongsawatdigul, 2013; Li et al., 2006). Previous studies have shown that mungbean hydrolysed using bromelain could generate flavour peptides that have umami flavour characteristics and also antioxidant properties (Sonklin, Laohakunjit & Kerdchoechuen, 2011; Sonklin et al., 2018). Despite the antioxidant properties from mungbean meal protein hydrolysate have been reported, the capability of the peptide fractions to retain these activities is unknown. Therefore, the objectives of this study were to optimize the conditions used to generate mungbean meal protein hydrolysate with antioxidant activity from bromelain and to investigate the antioxidant activities of each peptide fractionated based on different molecular weight.

## Materials and Methods

### Materials

Mungbean meal and bromelain was provided from Sittinun Co., Ltd., Thailand and K-Much-Industry Co., Ltd. (Bangkok, Thailand). Mungbean meal was defatted using hexane based on a previously described method (Sonklin, Laohakunjit & Kerdchoechuen, 2011). All chemical reagents were of analytical grade. 2,2-diphenyl-1-picrylhydrazyl (DPPH), glutathione reduced (GSH), 1-10-Phenanthroline, 2,4,6-tris(2-pyridyl)-s-triazine (TPTZ), 3-(2-pyridyl)-5,6-diphenyl-1,2,4-triazine-p,p′-disulfonic acid monosodium salt hydrate (ferrozine) and pyrogallol were purchased from Sigma Aldrich (MO, USA). All other reagents were purchased from Fisher Scientific (Hampton, NH, USA) except stated otherwise.

### Screening conditions to produce enzymatic MMPH using Response Surface Methodology (RSM)

Response Surface Methodology (RSM) was used for screening the optimal conditions used to produce mungbean protein hydrolysate according to the method described by Sonklin et al. (2018). The optimal condition was selected based on enzyme content and hydrolysis time which contributed to the highest degree of hydrolysis, DPPH and ABTS scavenging activities. Defatted mungbean meal 10 g was mixed in 100 mL of distilled water, and acidified to pH 6.0 using 2 M HCl. Bromelain was added to samples at 5, 10, 15 and 20% (w/w). All samples were hydrolysed for 6, 12, 18 and 24 h, respectively. After complete reaction at each hydrolysis time, enzymatic reaction was terminated by heating at 95 °C for 15 min, cooled in a water bath, filtered, freeze dried and analysed for degree of hydrolysis (DH) (Flavia & Maria, 1998), DPPH and ABTS (Sonklin et al., 2018) as bellowed.

*The degree of hydrolysis (DH)*: The DH was determined using trichloroacetic acid (TCA), as described by Flavia & Maria (1998). Total protein was measured by the Kjeldahl method. The DH values were calculated with the following equation.

\begin{eqnarray*}\text{%}DH=(\text{Soluble protein in TCA}/\text{Total protein})\times 100. \end{eqnarray*}

*DPPH scavenging activity*: The scavenging activity of peptide samples against DPPH radicals was determined using a previously described method (Girgih et al., 2011) with slight modifications for a 96-well microplate. Samples were dissolved in 0.1 M sodium phosphate buffer, pH 7.0 containing 1% (w/v) Triton X-100. DPPH was dissolved in methanol to a final concentration of 100 µM. Peptide samples (100 µL) were mixed with 100 µL of the DPPH solution in the 96-well microplate to a final assay concentration of 0.5 mg/mL and incubated at room temperature in the dark for 30 min. The absorbance values of the blank (A_b_) and samples (A_s_) were measured at 517 nm. The blank well contained sodium phosphate buffer instead of the peptide sample. The percentage of DPPH radical scavenging activity of the samples was determined using the following equation:

\begin{eqnarray*}\text{DPPH radical scavenging activity}(\text{%})= \frac{({\mathrm{A}}_{\mathrm{b}}-{\mathrm{A}}_{\mathrm{s}})}{{\mathrm{A}}_{\mathrm{b}}} \times 100. \end{eqnarray*}

*The ABTS radical-scavenging activity*: The ABTS activity was determined with an assay, modified from Binsan and co-workers (2008). The stock solutions were 7.4 mM 2,2′-azino-bis (3-ethylbenzthiazoline-6-sulfonic acid) (ABTS) solution and 2.6 mM potassium persulfate solution. The working solution was prepared by mixing the two stock solutions in equal quantities and allowing them to react for 12 h at room temperature in the dark. The solution was then diluted by mixing 1 mL ABTS solution with 50 mL ethanol to obtain an absorbance of 1.1 ± 0.02 units at 734 nm using a spectrophotometer. Fresh ABTS solution was prepared for each assay. The sample (50 µL) was mixed with 950 µL of ABTS solution, and the mixture was left at 25 °C for 2 s in the dark. The absorbance was then measured at 734 nm using a spectrophotometer. The control was prepared in the same manner, except distilled water was used instead of the sample. The blank was 50 µL of sample solution mixed with 950 µL ethanol. The ABTS radical-scavenging activity was calculated as:

\begin{eqnarray*}\text{ABTS radical scavenging activity}(\text{%})= \frac{({A}_{\mathrm{control}}+{A}_{\mathrm{blank}})-{A}_{\mathrm{sample}}}{{A}_{\mathrm{control}}} \times 100. \end{eqnarray*}

For the RSM step, enzyme concentration (*x*_1_) and hydrolysis time (*x*_2_) were independent variables. Three responses (*y*) were analysed DH, DPPH and ABTS. Each response can be described by the equation below (Khuri & Cornell, 1987).

\begin{eqnarray*}y={b}_{0}+{b}_{1}{x}_{1}+{b}_{2}{x}_{2}+{b}_{12}{x}_{1}{x}_{2}+{b}_{11}{x}_{1}^{2}+{b}_{22}{x}_{2}^{2} \end{eqnarray*}

Where: *b*_0_ was the constant regression coefficients of the model. *b*_1_ and *b*_2_ were the linear regression coefficients of the model. *b*_11_ and *b*_22_ were the quadratic regression coefficients of the model. *b*_12_ was the interaction regression coefficients.

The experiment plan was designed and statistically analysed for the effects of the independent variables using the SPSS 22.0 program. A *p*-value less than 0.05 was considered statistically significant (*p* < 0.05). Statistica program 5.0 (StatSoft Inc., 1995, USA) was used to build the surface plots of each variables.

### Preparation of peptide fractions with different molecular weight distributions

MMPH (crude-MMPH) from the optimized hydrolysis condition was selected for the ultrafiltration step. The crude-MMPH was fractionated through a series of ultrafiltration membranes with molecular weight cut-off (MWCO) of 10, 5 and 1 kDa (Millipore, Darmstadt, Germany). This process yielded four fractions: F1 (MW > 10 kDa), F2 (MW 5–10 kDa), F3 (MW 1–5 kDa) and F4 (MW <1 kDa). The fractions were freeze-dried and stored at −20°C until used.

### Determination of the amino acid compositions

Total and free amino acid of crude-MMPH and free amino acid of peptide fractions (F1, F2, F3, and F4) were analysed according to the method described by Li et al. (2008). Pre-treatment of total amino acid analysis, sample was carried out by hydrolysing samples using 6 M HCl at 110 °C for 22 h, samples were vigorously shaken and filtered. The acid in the permeate dried using a desiccator and re-dissolved using 1 mL of 0.02 HCl. The dried samples were kept at 4 °C until it was injected into the HPLC. For the pre-treatment of free amino acid analysis, trichloroacetic acid (TCA) was added to samples at ratio 1:1 and incubated for 2 h. After incubation, samples were filtered and centrifuged at 6,000 g for 10 min. Supernatant was collected and stored at 4 °C before injection. After pre-treatment step, each samples were injected into reversed-phase, high performance liquid chromatography (RP-HPLC) (Agilent 1100; Agilent Technologies, Santa Clara, CA, USA). The following conditions were used for analysis. O-phthaldialdehyde (OPA) was added into column (Zorbax 80 A C18 column (4.6 i.d. × 180 mm; Agilent Technologies, Palo Alto, CA, USA)) for pre-column derivatization. Samples (1 µL) were injected into the column using two mobile phases. Mobile phase A was 7.35 mM of sodium acetate : triethylamine : tetrahydrofuran at ratio 500 : 0.12 : 2.5 (v/v/v) and acidified to pH 7.2 with acetic acid. Mobile phase B was 7.35 mM sodium acetate : methanol : acetonitrile with ration 1:2:2 (v/v/v) and adjusted to pH 7.2. The system was operated at 40° C and peaks detected at two wave-lengths 338 and 262 nm. Eighteen amino acids (Ala, Arg, Asp, Cys, Glu, Gly, His, Ile, Leu, Lys, Met, Phe, Pro, Ser, Thr, Tyr, Val and Trp) were used as external standards.

### Antioxidant properties of peptide fractions

#### DPPH scavenging activity

The scavenging activity of peptide samples against DPPH radical was determined using the described method of Girgih et al. (2011) which was explained in above. The EC_50_ (peptide fractions that inhibited 50% of DPPH radical) of the samples were determined. The EC_50_ was calculated using a non-linear regression from a plot of percentage of DPPH activity versus peptide concentrations (0.2, 0.4, 0.6, 0.8 and 1.0 mg/mL). Glutathione, which was positive control (glutathione, GSH), was reacted with DPPH as same as samples.

#### Hydroxyl radical scavenging activity

The hydroxyl radical scavenging assay was modified using the method described by Ajibola et al. (2011). Samples and positive control (glutathione, GSH) were dissolved in 0.1 M sodium phosphate buffer (pH 7.4) to a final concentration of 2 mg/mL. The reactions were carried out in a 96-well microplate. Fifty microliters of samples or buffer (Blank) was mixed with 50 µL of 3 mM 1, 10-phenanthroline in 0.1 M sodium phosphate buffer (pH 7.4) and 50 µL of 3 mM FeSO_4_. To initiate the Fenton reaction, 50 µL of 0.01% hydrogen peroxide (H_2_O_2_) was added and absorbance read at 536 nm every 10 min for 1 h while incubating at 37 °C with continuous shaking. The percentage hydroxyl radical scavenging activity was calculated using the following equation:

\begin{eqnarray*}\text{Hydroxyl radical scavenging activity}(\text{%})\nonumber\\\displaystyle \quad = \frac{(\Delta {\text{Amin}}^{-1} \left( \text{blank} \right) -\Delta {\text{Amin}}^{-1}(\text{sample}))}{\Delta {\text{Amin}}^{-1} \left( \text{blank} \right) } \times 100 \end{eqnarray*}

where $\Delta {\text{Amin}}^{-1} \left( \text{blank} \right) $ and ΔAmin^−1^(sample) were the change in rate of reaction of blank and sample, respectively.

#### Superoxide radical scavenging activity

A range of sample concentrations (0.2, 0.4, 0.6, 0.8 and 1.0 mg/mL final) were prepared by dissolving them in 50 mM Tris-HCl buffer, pH 8.3 containing 1 mM EDTA. Samples (80 µL) were pipetted into clear microplate well, buffer was used for the blank well. Glutathione (GSH) was used as the positive control. The reaction was measured immediately using a spectrophotometer at 420 nm after adding 40 µL of 1.5 mM pyrogallol in 10 mM HCL for 4 min at intervals of 1 min (Xie et al., 2008). The superoxide scavenging activity was calculated using the following equation:

\begin{eqnarray*}\text{superoxide scavenging activity}(\text{%})= \frac{(\Delta {\text{Amin}}^{-1} \left( \text{blank} \right) -\Delta {\text{Amin}}^{-1}(\text{sample}))}{\Delta {\text{Amin}}^{-1}(\text{blank})} \times 100 \end{eqnarray*}

where $\Delta {\text{Amin}}^{-1} \left( \text{blank} \right) $ and $\Delta {\text{Amin}}^{-1} \left( \text{sample} \right) $ were the change in rate of reaction of blank and sample, respectively.

### Ferric reducing antioxidant power (FRAP)

The FRAP assay was performed according to the method of Benzie & Strain (1996) as described by Karamać and co-worker (2016). The FRAP reagent was prepared by mixing 0.3 M acetate buffer, 10 mM TPTZ at pH 3.6 in 40 mM HCl, 20 mM FeCl_3_.6H_2_O pH 3.6 at ratio of 5:1:1 (v/v/v). Samples were dissolved in deionized water at concentration of 2 mg/mL. The reaction was carried out in a 96-well microplate. Samples and FRAP reagent were incubated at 37 °C before adding 40 µL of samples to 200 µL of the FRAP reagent and absorbance was measured at 593 nm. Glutathione (GSH), which was the positive control, was reacted with FRAP reagent as same as sample. A standard curve was generated using FeSO_4._7H_2_O (0.03–0.9 µmol/mL) and results were reported as mmole of Fe^2+^ reduced per g protein from the regression slope of the standard curve.

#### Metal ion chelation activity

The metal ion chelating activity of peptide fractions was measured using the method described by Xie et al. (2008). Samples were prepared to final concentrations of 0.5, 1.0, 3.0 and 5.0 mg/mL using the deionized water, which served as the blank. Samples and glutathione (GSH, positive control) were separately mixed with 0.05 mL of 2 mM FeCl_2_, 1.85 mL of deionized water, 0.1 mL of 5 mM of ferrozine solution in a reaction tube for 10 min at room temperature. Subsequently, 20 µL of mixed solution were pipetted into 96 well microplates and absorbance measured at 562 nm. The percentage of chelating activity was calculated using the following equation:

\begin{eqnarray*}\text{Metal chelating activity}(\text{%})= \frac{({\mathrm{A}}_{\mathrm{b}}-{\mathrm{A}}_{\mathrm{s}})}{{\mathrm{A}}_{\mathrm{b}}} \times 100. \end{eqnarray*}

Where A_b_ is the absorbance values of blank, and A_s_ is the absorbance values of sample.

### Statistical analysis

All assays were determined in triplicate. Results were subjected to one way analysis of variance (ANOVA) using SAS program Version 6.0 (SAS Institute, 1997, USA). Statistical significance of difference between samples was accepted at *p*-value less than 0.05 (*p* < 0.05) using the Duncan’s multiple range test (DMRT).

## Results

### Screening of optimum conditions to produce MMPH by bromelain using RSM

The optimum bromelain treatment conditions used to produce MMPH was determined using the RSM. The effect of different enzyme treatment conditions on DH, DPPH and ABTS are reported using a quadratic equation. Enzyme concentration (*x*_1_) and hydrolysis time (*x*_2_) a statistically significant interaction (*p* < 0.05) on the DH, DPPH and ABTS value were shown in Table 1 and significant values of DH, DPPH and ABTS were performed in the equations for each variable are shown below:

(1) \begin{eqnarray*}& & DH:y=8.401+3.305{X}_{1}+2.318{X}_{2}-0.0986{X}_{1}^{2}-0.0654{X}_{2}^{2}-0.0256{X}_{1}{X}_{2}\end{eqnarray*}

(2) \begin{eqnarray*}& & DPPH:y=-2.967+8.468{X}_{1}+3.429{X}_{2}-0.254{X}_{1}^{2}-0.0951{X}_{2}^{2}-0.0307{X}_{1}{X}_{2}\end{eqnarray*}

(3) \begin{eqnarray*}& & ABTS:y=0.238+9.255{X}_{1}+1.418{X}_{2}-0.310{X}_{1}^{2}-0.0296{X}_{2}^{2}--0.0007961{X}_{1}{X}_{2}.\end{eqnarray*}

**Figure 1:**
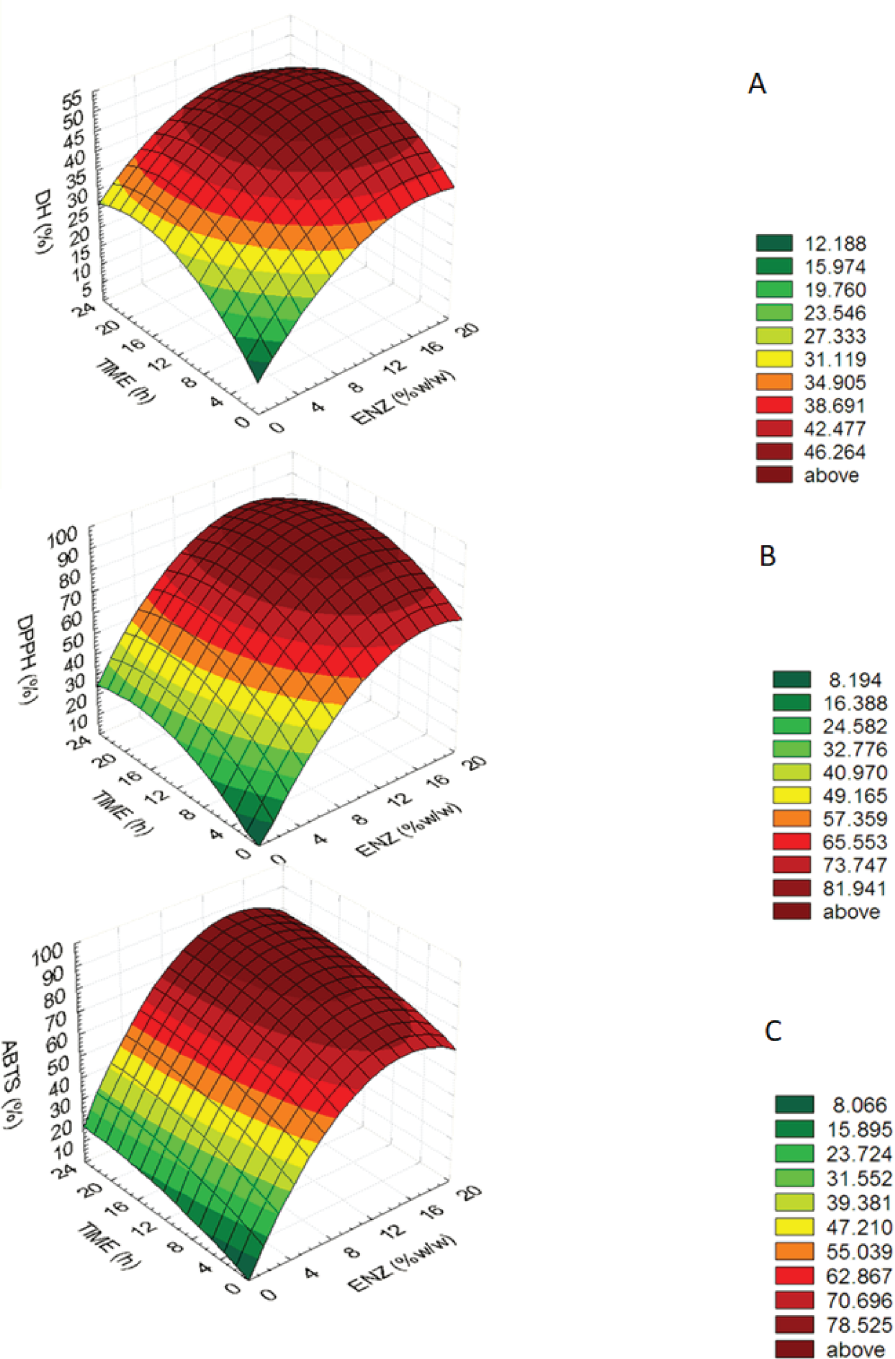
Response surface plots for the effects of enzyme concentration and hydrolysis time. Response surface plots for the effects of enzyme concentration and hydrolysis time on (A) the degree of hydrolysis (DH), (B) DPPH radical-scavenging activity (DPPH) and (C) ABTS radical-scavenging activity (ABTS).

The equations showed that enzyme concentration (*X*_1_) was higher important factors affecting DH, DPPH and ABTS values than hydrolysis time (*X*_2_) because an estimated regression coefficient of *X*_1_ presented the higher value. The determination coefficients (*R*^2^) of the DH, DPPH and ABTS were 0.818, 0.925 and 0.969, respectively. The equations were used to build response surface plots to predict the critical points and effectiveness of DH (Fig. 1A), DPPH (Fig. 1B) and ABTS (Fig. 1C). For response surface plots, DH, DPPH, ABTS values increased with increasing enzyme concentration and hydrolysis time until the critical point, all of three variables were constant and slightly decreased. Results indicated that enzyme concentration and hydrolysis time had influenced on DH, DPPH, ABTS values of mungbean meal protein hydrolysate.

**Table 1:** Model Regression Coefficients Estimated by Multiple Linear Regressions for the degree of hydrolysis (DH), DPPH radical-scavenging activity (DPPH) and ABTS radical-scavenging activity (ABTS) of MMPHs.^a^

Factor	Coefficient		
	DH	DPPH	ABTS
constant	8.401^*^	−2.967^*^	0.238^*^
linear			
*X*_1_	3.305^*^	8.468^*^	9.255^*^
*X*_2_	2.318^*^	3.429^*^	1.418^*^
quadratic			
${X}_{1}^{2}$	−0.0986^*^	−0.254^*^	−0.310^*^
${X}_{2}^{2}$	−0.0654^*^	−0.0951^*^	−0.00296^*^
interactions			
*X*_1_ × *X*_2_	−0.0256^*^	−0.3070^*^	0.0007961^*^
*R*^2^	0.818	0.925	0.969

**Notes.**

a A model in which *x*_1_ =  enzyme concentration, *x*_2_ =  hydrolysis time.

* Significant at *p* < 0.05.

**Figure 2:**
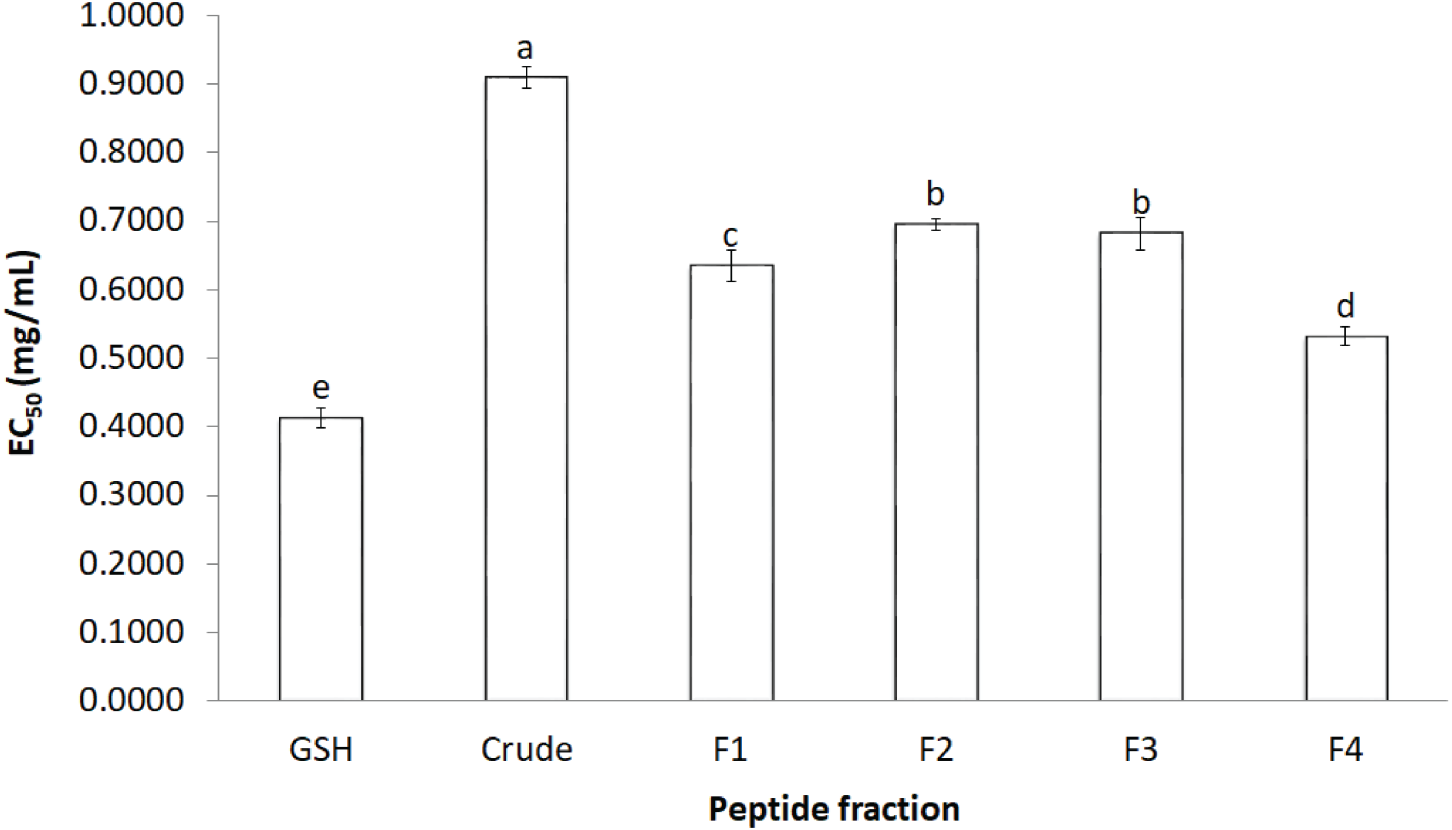
The effective concentration that scavenged 50% (EC_50_) values for DPPH. The effective concentration that scavenged 50% (EC_50_) values for DPPH scavenging activity values of crude-MMPH and its ultrafiltrated peptide fraction.

**Figure 3:**
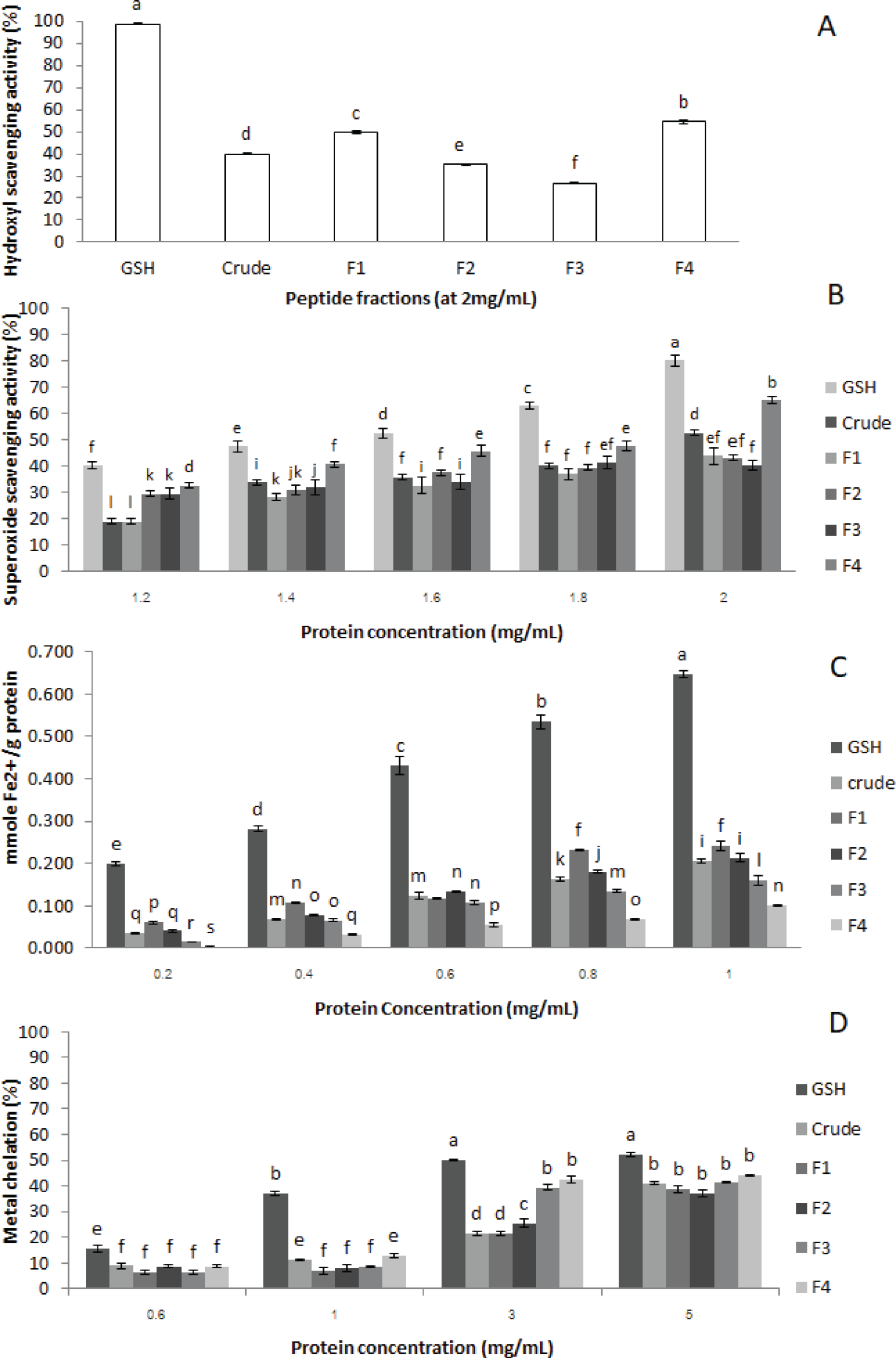
Antioxidant activities of crude-MMPH and its ultrafiltrated peptide fraction. (A) hydroxyl scavenging activity, (B) superoxide scavenging activity, (C) ferric reducing antioxidant power (FRAP), and (D) metal chelation activity.

An optimum condition to produce MMPH was selected from an overlay of Figs. 1–3 and choosing the range of enzyme concentration and hydrolysis time which gave the maximum DH, DPPH and ABTS activity. Results showed that the optimal enzyme concentration was 10–18% and hydrolysis time 8–24 h. Therefore, 15% of bromelain and 12 h hydrolysis time were chosen to produce MMPH. The models used were confirmed by observed and predicted values as showed in Table 2. Results showed that the predicted values which were derived from the models were close to the observed value.

**Table 2:** Observed and predicted values for optimizing the hydrolysis condition.

**Trial**	**Conditions**^**a**^	**DH**		**DPPH**		**ABTS**	
		**obs**	**pre**	**obs**	**pre**	**obs**	**pre**
1	*x*_1_ = 10%, *x*_2_ = 18 h	48.9	47.5	79.1	81.7	81.2	77.9
2	*x*_1_ = 15%, *x*_2_ = 12 h	46.7	49.6	94.9	88.8	82.3	82.5
3	*x*_1_ = 15%, *x*_2_ = 24 h	40.1	44.3	78.4	83.4	82.2	86.6
4	*x*_1_ = 20%, *x*_2_ = 12 h	45.0	47.3	86.4	84.9	77.1	77.6
5	*x*_1_ = 20%, *x*_2_ = 24 h	43.1	40.8	79.0	77.6	78.0	78.7

**Notes.**

obs observed pre predicted

^a^ *x*_1_ represents enzyme concentration. *x*_2_ represents hydrolysis time.

### Amino acid composition of peptide fractions

The crude-MMPH was analysed for both total amino acids and free amino acids, but F1, F2, F3 and F4 were used to analyse only total amino acids. High content of Glu, Asp, Lys, Arg, Leu, Ser, Pro and Phe were found in the crude-MMPH (162.66, 104.39, 72.11, 67.00, 62.93, 54.27, 48.01 and 45.61 mg/g protein, respectively) (Table 3). The predominant amino acids were hydrophobic amino acids (HHA) group. The highly abundant in peptides were found in Crude-MMPH. Therefore, it can refer that F1-F4 had a plenty of peptides as well. Asp and Glu were the highest in all peptide fractions (Table 3). In particular, F4 had the highest content of Glu, Arg, Gly, Leu, Met, Tyr, Phe, Trp and Ser. All peptide fractions had high amounts of total HAA, and the contents were quite similar in each fraction. The proportion of aromatic amino acid contents (Tyr, Phe, Trp) in fraction F4 was also found to be the highest compared to the other peptide fractions.

**Table 3:** Amino acid composition of crude-MMPH and its ultrafiltrated peptide fractions.

	**Content (mg / g Protein)^a^**				**Total amino acid(% w/w Protein)^a^**			
**Group**	**Amino acid**	**Total amino acids**	**Free amino acids**	**Amino acid in peptide form**	**F1**	**F2**	**F3**	**F4**
Acidic (−)	Asp	104.39 ± 3.93	1.06 ± 0.04	103.33 ± 3.89	14.23 ± 0.15	13.84 ± 1.54	13.74 ± 0.57	11.77 ± 0.80
	Glu	162.66 ± 2.044	16.92 ± 0.51	145.70 ± 2.55	18.30 ± 0.02	17.95 ± 0.23	18.23 ± 0.95	18.72 ± 0.95
Basic (+)	Lys	72.11 ± 6.12	28.94 ± 0.20	43.17 ± 5.93	8.15 ± 0.19	8.32 ± 0.43	8.00 ± 0.64	6.89 ± 0.45
	Arg	67.00 ± 1.39	22.10 ± 0.27	44.91 ± 1.12	6.63 ± 0.57	6.71 ± 0.55	6.72 ± 0.26	6.85 ± 0.47
	His	20.01 ± 0.76	11.58 ± 0.59	8.81 ± 0.16	3.55 ± 0.66	3.07 ± 0.69	2.75 ± 0.55	3.02 ± 0.56
Hydrophobic	Gly	36.78 ± 2.52	5.49 ± 0.86	31.29 ± 3.38	3.19 ± 0.45	3.78 ± 0.48	3.73 ± 1.07	3.98 ± 0.20
	Ala	40.02 ± 1.26	10.36 ± 0.23	29.66 ± 1.03	4.12 ± 0.28	3.97 ± 1.03	3.91 ± 0.80	4.88 ± 0.57
	Val	36.48 ± 1.50	6.68 ± 0.25	29.80 ± 1.75	4.48 ± 0.54	5.18 ± 0.45	5.30 ± 0.45	5.02 ± 0.95
	ILe	36.86 ± 1.84	3.63 ± 0.17	33.23 ± 2.01	4.72 ± 0.55	4.64 ± 0.69	4.67 ± 2.06	3.56 ± 0.95
	Leu	62.93 ± 0.15	22.29 ± 0.14	40.64 ± 0.11	6.48 ± 0.28	6.63 ± 0.91	6.70 ± 0.74	8.60 ± 0.74
	Pro	48.01 ± 1.09	3.57 ± 0.10	44.44 ± 0.99	4.97 ± 0.22	5.59 ± 1.19	6.17 ± 0.26	3.62 ± 0.71
	Met	8.90 ± 1.39	4.60 ± 0.11	4.30 ± 1.50	1.00 ± 0.21	1.13 ± 0.37	1.09 ± 0.49	1.60 ± 0.54
	Cys	3.97 ± 0.34	0.19 ± 0.23	3.78 ± 0.57	2.27 ± 0.18	0.83 ± 0.64	0.60 ± 0.54	0.30 ± 0.16
Aromatic	Tyr	29.03 ± 2.72	5.24 ± 1.15	23.79 ± 1.58	3.73 ± 0.08	3.37 ± 0.62	3.44 ± 0.57	3.81 ± 0.57
	Phe	45.61 ± 1.39	7.06 ± 0.16	38.55 ± 1.54	4.73 ± 0.23	5.08 ± 0.43	5.17 ± 1.78	6.58 ± 0.93
	Trp	6.05 ± 0.31	1.18 ± 0.17	4.87 ± 0.48	0.70 ± 0.04	0.74 ± 0.40	0.78 ± 0.76	0.83 ± 0.70
Hydrophilic	Ser	54.27 ± 2.38	13.70 ± 0.88	40.57 ± 3.25	5.48 ± 0.29	5.76 ± 1.03	5.67 ± 0.79	6.50 ± 0.76
	Thr	33.41 ± 1.71	5.93 ± 0.27	27.48 ± 1.44	3.34 ± 0.35	3.46 ± 0.33	3.37 ± 0.06	3.52 ± 0.76
Total		868.51	170.54	697.98	100	100	100	100

**Notes.**

a Each value is expressed as the mean ± SD (*n* = 3).

#### DPPH radical scavenging activity

The activity of crude-MMPH and its ultrafiltrated peptide fractions to scavenge the DPPH radical is presented as the EC_50_ value (Fig. 2). The *R*^2^ of GSH, Crude-MMPH, F1, F2, F3 and F4 was 0.9922, 0.9771, 0.8888, 0.9805, 0.9619 and 0.7962, respectively. Crude-MMPH and its peptide fractions scavenged the radical to a 50% inhibition at a range of 0.4 –0.9 mg/mL. All peptide fractions had low EC_50_ values of DPPH than the hydrolysate. EC_50_ value of fraction 4 showed the lowest EC_50_ as 0.53 mg/mL, followed by F1, F3 and F2 with EC_50_ as 0.63, 0.68 and 0.69, respectively. EC_50_ value of fraction 4 (0.53 mg/mL) was quite close to the EC_50_ value of standard which was glutathione (0.41 mg/mL).

#### Hydroxyl radical scavenging activity

Hydroxyl radicals scavenging activity of crude-MMPH and its ultrafiltrated peptide fractions were analysed in triplicates. Crude-MMPH and its fractions exhibited different hydroxyl scavenging activities, as shown in Fig. 3A. The <1 kDa (F4) fraction had superior ability when compared to crude-MMPH and all other fractions. F4 showed 54.50 % of hydroxyl scavenging activity, followed by F1, crude-MMPH, F2 and F3 which were 49.64, 39.91, 35.18 and 26.61, respectively. Glutathione which was a standard showed the significantly greater than Crude-MMPH and its ultrafiltration peptide fractions (98.68%).

#### Superoxide radical scavenging activity

Superoxide radicals scavenging activity assay was used to determine the antioxidant activity of crude-MMPH and peptide fractions. Figure 3B shows that the superoxide radical scavenging activity of crude-MMPH and its fractions increased distinctly with increasing concentration from 1.2 to 2.0 mg/mL. The peptide fraction less than 1 kDa (F4) showed stronger superoxide scavenging activity in all concentration in comparison to the peptide fractions that had higher molecular weight. At 1.8 mg/mL of peptide concentration, F4 contributed to the greatest superoxide radical scavenging activity with 65.10%. The superoxide radical scavenging activity of GSH showed 63.06% which was slightly higher than F4. Even though samples were used at 2 mg/mL of protein concentration, the superoxide radical scavenging activity of all samples was lower than 50%.

### Ferric reducing activity power (FRAP)

FRAP is related to the generated Fe^2+^ as shown in mmole Fe^2+^/g protein. As depicted in Fig. 3C, FRAP of crude-MPH and its fraction increased significantly with increasing protein concentration from 0.2–1 mg/mL. Moreover, FRAP of peptide fractions in this study increased with increasing in the molecular weight of peptides. F1 (>10 kDa), which had a HMW, had the greatest reducing power at all concentrations. F1 generated Fe^2+^ as 0.061, 0.107, 0.118, 0.231 and 0.242 mmole Fe^2+^/g protein when used protein concentration at 0.2, 0.4, 0.6, 0.8 and 1.0 mg/mL protein, respectively.

#### Metal chelation activity

Metal chelation activity of crude-MMPH and all ultrafiltrated peptide fractions had poor activity. At the highest concentrations (5 mg/mL), metal chelation activity value of all peptide fractions was less than 50% (Fig. 3D). Glutathione showed a low metal chelation activity (53.25 %) as well. In addition, the current study showed that low molecular weight peptide fractions contributed good metal chelation activity of all concentrations. Particularly, the highest metal chelation activity was observed for only F4. F4 fraction contributed to the greatest activity with 43.94% when using concentration at 5 mg/mL. The metal chelation activity of F3 and F4 at 5 mg/mL did not significant difference from 4 mg/mL. Results can identify that 5 mg/mL was the limited concentration of MMPH peptides for metal chelation activity. Although the metal chelation activity of other peptide fractions was inconsistent, the trend was slightly increased with decreased molecular weights.

## Discussion

The optimum condition to produce MMPH was investigated using response surface plots of DH, DPPH and ABTS which were built by statistically acceptable equations at *p* < 0.05. Because the determination coefficients (*R*^2^) of the DH (0.818), DPPH (0.925) and ABTS (0.969) equations were higher than 0.7, which indicated that the regression models were appropriate and acceptable (Moore, Notz & Flinger, 2013). In addition, the predicted values were close to the observed value (Table 1). At the end of DH, DPPH and ABTS response surface plots (Figs. 1A–1C), they showed constant and slightly decreased. It might be cause of an over saturation of the protease enzyme. The over saturation of the protease enzyme occurred when using higher enzyme concentration and hydrolysis time, brought about decreasing in hydrolysis which caused a decrease in its biological activity. The DPPH and ABTS value were also decreased (Ovissipour et al., 2009).

Amino acid composition of crude-MMPH which derived from the optimal condition was analysed. Crude-MMPH contained highly abundant in hydrophobic amino acids (HAA) because bromelain which is an endoprotease which cleaved at hydrophobic position inside the protein chain. Especially, Arg, Lys and Phe were the site-specific cleavage of bromelain enzyme, resulting in high amount of HAA (Adler-Nissen, 1986; Sonklin, Laohakunjit & Kerdchoechuen, 2011). F4 had the highest proportion of aromatic and hydrophobic amino acids in peptide chain compared to other peptide fractions. Moreover, F4 contained low molecular weight peptide less than 1 kDa. Therefore, it can be assumed that F4 will exhibit the highest antioxidant activity. From the previous studies, LMW peptides which were rich in aromatic and hydrophobic amino acids in chain expressed high antioxidant potentials (Ajibola et al., 2011).

In order to confirm the antioxidant abilities and to investigate type of antioxidant reaction of peptides from mungbean meal protein hydrolysate. Five antioxidant activities were used to analyse. All ultrafiltrated peptide fractions had low EC_50_ values of DPPH than that of the hydrolysate. This indicated that purified peptides exhibited stronger antioxidant activity based on their molecular weight as the F4 (≤1 kDa) had a significantly lower EC_50_ value than other peptide fractions and crude-MMPH. The low molecular weight (LMW) peptides scavenged DPPH activity better than the high molecular weight (HMW) peptides. Peptide fractions from canola meal protein, barley hordein and hemp protein hydrolysate have shown similar activities and trends to those observed in this study (Alashi et al., 2014; Bamdad & Chen, 2013; Girgih et al., 2011). Glutathione (GSH) was used as a positive control in this study because GSH is a peptide which contributes to great antioxidant activity. GSH composed of cysteine, glutamic acid glycine in its peptide chain and it can be antioxidant by serving as a proton donor to free radicals (Pompella et al., 2003). Samples in this study were also peptide which had high abundance of glutamic amino acid and hydrophobic amino acid in peptide chain. As the basic knowledge, the hydrophobic can inhibit free radicals by proton donation. Therefore, the properties of glutathione can be a proper standard for peptide samples in this study. Moreover, EC_50_ value of F4 was close to the glutathione. It implied that the peptide fraction of MMPH have molecular weight less than 1 kDa was an effective antioxidant peptides which could scavenge DPPH radical and prevent the free radical associated with oxidative stress. The peptide in mungbean meal protein hydrolysate can donate hydrogen atom to DPPH radical. The reaction is based on a colour change from purple (DPPH) to clear yellow colour due to DPPH gaining the proton and becoming a stable radical molecule (Liu et al., 2010). Besides MW, the amino acid composition is an important factor that affects antioxidant properties. Peptide fraction F4, which exhibited the highest DPPH scavenging activity, had high contents of total hydrophobic and aromatic amino acid. Peptides that are composed of these amino acids in their chain can easily scavenge the DPPH radical. In addition, the hydrophobic and aromatic group improved the solubility of peptide in non-polar environment (Kim, Je & Kim, 2007). Therefore, mungbean meal peptides showed strong inhibition of DPPH radicals.

Hydroxyl radicals are highly active when compared to other reactive oxygen radicals. The hydroxyl radical can react with almost all biomolecules in cells, affecting ageing and chronic diseases. Consequently, the defence of the hydroxyl radical is necessary for the protection of many metabolic disorders associated with the hydroxyl radical (Ajibola et al., 2011). The high activity observed for F4 may also be an influence of the low molecular weight peptides, as reported in various studies (Cheung et al., 2012; Ajibola et al., 2011). By contrast, F1 (>10 kDa) showed higher hydroxyl radical scavenging activity than F2 (5–10 kDa) and F3 (1–5 kDa) that they had lower molecular weight than F1. From this study, it could be indicated that amino acid composition in peptide chain had more influenced on hydroxyl scavenging activity than size of peptide. However, sequence in peptide might have strong effected on activity as well.

MMPH peptides and GSH (positive control) showed low hydroxyl radical scavenging activity. It might be a reason that MMPH peptides had poor ability to react with hydroxyl radical by donating electron or form double bond, in order to become hydroxycyclohexadienyl radicals which are stable molecules (Lee, Koo & Min, 2004). Results correlated with amino acid composition in peptide chain of mungbean meal protein which composed of higher amount of hydrophobic amino acid than Met, Cys and aromatic amino acid which Met, Cys and aromatic amino acid could donate electron and form double bond to hydroxyl radical (Lee, Koo & Min, 2004). Therefore, peptides from mungbean meal protein showed low activity even using concentration to 2 mg/mL. In addition, crude-MMPH expressed higher activity than its purify peptides which were F2 and F3. It might be due to the potential synergistic effect of various peptides in crude-MMPH.

Another harmful oxygen specie is superoxide radicals (O${}_{2}^{{^{\prime}}{^{\prime}}}$) because a lot of biological reactions can produce the superoxide radicals. Superoxide radicals are very important precursor to generate various highly reactive radicals such as hydroxyl radical and can be the necessary radical for the initiation of lipid oxidation. Therefore, superoxide radicals are used to determine the antioxidant activity of hydrophilic and hydrophobic compounds (Kanatt, Chander & Sharma, 2007). From the results in this study, low molecular weight peptides showed the greatest superoxide radical scavenging activity. It indicated that had influence on superoxide activity. Even though mungbean protein peptide fractions composed of high content of Glu and Asp which they were reported that contribute strong inhibition of superoxide radicals (Tao et al., 2018), this peptide fractions did not show high superoxide radical scavenging activity. The cause for the low superoxide activity was not clear, but it may be related to sequence of Glu, Asp and Arg in side chain. These amino acids contribute to strong activity when they were in the terminal of chain because they could donate electron to superoxide radical (Tao et al., 2018). Thus, results could predict that most of mungbean peptide might be Glu, Asp and Arg were in internal chain. More interesting aspect from this study, this is evident in the observed activity as superoxide radical scavenging activity slightly higher than those of the hydroxyl radical scavenging activity at 2 mg/mL, whereas the hydroxyl radicals are more potent reactive species than the superoxide radicals (Cacciuttolo et al., 1993). Results indicated that peptides from mungbean meal were good ability for suppression of superoxide radicals. However, results obviously indicated that MMPH is antioxidant peptide which moderate ability to prevent oxygen species radical (hydroxyl and superoxide) for cell damage protection.

FRAP assay is used to detect the ability of antioxidant substances to reduce ferric ions (Fe^3+^) to ferrous ions (Fe^2+^). The results are presented as Fe^2+^ reducing power which is the ability of the peptides to be electron donors and to stop the chain reaction that leads to the continuous production of free radicals (Carrasco-Castilla et al., 2012). This study showed that FRAP activity significantly increased with increasing of concentration from 0.2 mg/mL to 0.8 mg/mL and after that the activity of all peptide fractions was quite constant at 1.0 mg/mL. It might be a limit of ability of peptide to donate electron to Fe^3+^. In addition, FRAP of peptide fractions in this study increased with increases in the molecular weight of peptides. This implied that the molecular weight might not be the most important contributing factor to the ferric ion reducing power. The amino acid in peptide chain and sequence had also influence on FRAP antioxidant activity.

Result correlates with amino acid composition. Asp (acidic), Lys (basic), Ile (hydrophobic), Pro (hydrophobic) and Cys (aromatic) in F4 were found low amount compared to other peptide fractions. These amino acids play an important role for FRAP (Udenigwe & Aluko, 2011), leading to low FRAP activity in F4 (<1 kDa). Moreover, Met, Cys, Tyr, Met, Trp, His and Lys can improve ferric reducing potential (Rajapakse et al., 2005; Carrasco-Castilla et al., 2012) which these amino acids were lower in F4 had in comparison to the other peptide fractions. This might be one of the contributing factors affecting the reduction in ferric reducing activity power observed for F4.

Chelation of metal ions is an assay used to measure antioxidant ability, which the reactive oxygen species (ROS) are catalysed by transition metal iron and copper derived from chelation of metal ions. ROS stimulate the oxidation reaction of unsaturated lipid and promotion of oxidative damage at different levels (Saiga, Tanabe & Nishimura, 2003). The result from this study and some previous studies suggested that metal ion chelation activity may not depend on molecular weight, but might be associated with small molecule and the coordination site which can act as an iron chelator (Kong & Xiong, 2006). Results in this study show that mungbean meal peptides showed poor metal chelation activity as same as GSH (positive control). The reason might be related to high amount of hydrophobic amino acid in peptide chain which reacted with free radicals by proton donation. By contrast to metal chelation activity, this reaction related to electron donating substrates (Kong & Xiong, 2006). GSH reacted with free radical by proton donation, resulting in poor metal chelation activity in GSH as well. Moreover, Asp and Glu, especially at the end terminal of peptide chain, contributed to strong metal chelation activity. They contributed to extra electrons to improve electrostatic and ionic interaction between themselves and metal ion (Zhu et al., 2008). Even though mungbean peptide fractions were rich in Glu and Arg, peptides did not express high metal chelation activity. Results related to FRAP activity of mungbean peptides.

## Conclusion

MMPH and its ultrafiltration fractions had antioxidant activities with the potential to inhibit several antioxidant reactions. F4 that had molecular weight less than 1 kDa showed the highest DPPH radical scavenging activity, superoxide scavenging activity, hydroxyl scavenging activity and metal chelation activity, but this fraction had poor ferric reducing power. Moreover, the low molecular weight peptide fractions had antioxidant activities that were higher than the crude-MMPH, except for ferric reducing activity. This study could confirm that size of peptide and amino acid compositions in peptide chain have an important effect on antioxidant activities. From the mechanism of the reaction, this work determined the potential of antioxidant activity was divided into two main groups: proton donation (hydrogen atom transfer) and electron donation (electron transfer). The reaction of DPPH is proton donation. The reaction of FRAP, superoxide, hydroxyl is electron donation. Therefore, our findings suggested that the antioxidant mechanism of peptides obtained mungbean could react with many species of free radicals by multiple mechanisms, including donation of protons and electrons and chelation of transition metals. Moreover, mungbean meal peptides showed strong proton donation to free radicals (DPPH). The cause was that they were rich in hydrophobic amino acids and aromatic amino acids. However, to determine the specific mechanism of antioxidant activity, the sequences will be further studied to understand the correlation between the peptide structure and specific antioxidant mechanisms. This study shows that mungbean protein can be developed as a value-added commercial functional food ingredient, which it will increase its economic value.

##  Supplemental Information

10.7717/peerj.5337/supp-1 Supplemental Information 1 Raw data

## Additional Information and Declarations

### Data Availability

The following information was supplied regarding data availability: The raw data are provided in a Supplemental Information 1.
