# Supplementary material for: Assessment of antioxidant properties of membrane ultrafiltration peptides from mungbean meal protein hydrolysates
Source: PeerJ. 2018 Jul 27;6:e5337. doi: 10.7717/peerj.5337 (PMC6065462; doi:10.7717/peerj.5337)
Supplement: Supplemental Information 1 [file peerj-06-5337-s001.docx]

**Raw Data**

**Data Table 1**

**Raw data of DH, DPPH and ABTS derived from hydrolysis by bromelain with 0, 5, 10, 15, 20 % w/w and hydrolysis time 6, 12, 18, 24 hr**

| Enzyme concentration (%w/w) | Hydrolysis time (hr) | Rep. | DH | DPPH | ABTS |
| --- | --- | --- | --- | --- | --- |
|  |  |  |  |  |  |
| 0% | 6 | 1 | 22.04 | 21.64 | 12.65 |
|  |  | 2 | 20.46 | 22.45 | 12.53 |
|  |  | 3 | 21.25 | 23.05 | 12.65 |
|  | 12 | 1 | 24.70 | 20.94 | 10.58 |
|  |  | 2 | 24.64 | 21.14 | 11.39 |
|  |  | 3 | 25.71 | 20.74 | 11.27 |
|  | 18 | 1 | 25.00 | 20.94 | 12.99 |
|  |  | 2 | 25.69 | 19.93 | 13.11 |
|  |  | 3 | 23.98 | 20.03 | 12.99 |
|  | 24 | 1 | 20.93 | 20.54 | 12.88 |
|  |  | 2 | 22.57 | 19.93 | 12.30 |
|  |  | 3 | 22.29 | 19.93 | 13.45 |
| 5% | 6 | 1 | 34.88 | 40.97 | 44.86 |
|  |  | 2 | 33.71 | 41.38 | 52.43 |
|  |  | 3 | 34.51 | 41.68 | 45.66 |
|  | 12 | 1 | 45.93 | 64.23 | 45.43 |
|  |  | 2 | 46.65 | 80.94 | 45.55 |
|  |  | 3 | 47.69 | 78.72 | 45.55 |
|  | 18 | 1 | 47.26 | 47.11 | 57.64 |
|  |  | 2 | 47.91 | 58.99 | 58.50 |
|  |  | 3 | 46.92 | 76.51 | 58.04 |
|  | 24 | 1 | 45.80 | 70.77 | 61.94 |
|  |  | 2 | 43.83 | 69.06 | 63.09 |
|  |  | 3 | 46.72 | 57.89 | 62.29 |

**Raw data of DH, DPPH and ABTS derived from hydrolysis by bromelain with 0, 5, 10, 15, 20 % w/w and hydrolysis time 6, 12, 18, 24 hr (Cont.)**

| Enzyme concentration (%w/w) | Hydrolysis time (hr) | Rep. | DH | DPPH | ABTS |
| --- | --- | --- | --- | --- | --- |
| 10% | 6 | 1 | 38.37 | 42.08 | 66.30 |
|  |  | 2 | 38.86 | 61.41 | 66.30 |
|  |  | 3 | 37.61 | 77.32 | 67.10 |
|  | 12 | 1 | 42.55 | 73.99 | 78.68 |
|  |  | 2 | 41.28 | 75.81 | 81.77 |
|  |  | 3 | 42.90 | 94.63 | 83.26 |
|  | 18 | 1 | 48.91 | 76.51 | 81.54 |
|  |  | 2 | 47.89 | 79.03 | 81.31 |
|  |  | 3 | 49.90 | 81.85 | 80.97 |
|  | 24 | 1 | 41.05 | 85.67 | 82.12 |
|  |  | 2 | 41.08 | 86.98 | 81.43 |
|  |  | 3 | 42.38 | 74.19 | 82.23 |
| 15% | 6 | 1 | 44.62 | 66.14 | 66.53 |
|  |  | 2 | 43.04 | 87.79 | 66.64 |
|  |  | 3 | 44.72 | 90.91 | 66.18 |
|  | 12 | 1 | 45.75 | 93.62 | 81.09 |
|  |  | 2 | 48.06 | 94.83 | 81.43 |
|  |  | 3 | 46.11 | 96.34 | 84.41 |
|  | 18 | 1 | 44.05 | 83.36 | 87.28 |
|  |  | 2 | 46.82 | 79.23 | 76.96 |
|  |  | 3 | 46.11 | 76.41 | 77.30 |
|  | 24 | 1 | 41.06 | 70.47 | 83.15 |
|  |  | 2 | 40.32 | 81.04 | 81.09 |
|  |  | 3 | 39.03 | 83.66 | 82.46 |
| 20% | 6 | 1 | 49.29 | 85.87 | 72.37 |
|  |  | 2 | 50.65 | 78.32 | 75.35 |
|  |  | 3 | 51.31 | 80.64 | 76.84 |
|  | 12 | 1 | 49.36 | 95.03 | 77.65 |
|  |  | 2 | 49.65 | 73.59 | 75.47 |
|  |  | 3 | 50.29 | 90.60 | 77.42 |
|  | 18 | 1 | 48.38 | 78.52 | 77.76 |
|  |  | 2 | 47.09 | 81.24 | 76.96 |
|  |  | 3 | 46.09 | 89.50 | 76.50 |
|  | 24 | 1 | 45.40 | 86.07 | 77.07 |
|  |  | 2 | 40.93 | 71.38 | 79.37 |
|  |  | 3 | 43.02 | 79.63 | 77.53 |

**Data Table 2**

**DPPH scavenging activity values of crude-MMPH and its ultrafiltration peptide fraction.**

| Samples | concentration mg/mL | DPPH radical scavenging activity (%) | | |
| --- | --- | --- | --- | --- |
|  |  | Rep.1 | Rep.2 | Rep.3 |
| Crude-MMPH | 0.4 | 34.80 | 35.58 | 35.19 |
|  | 0.6 | 38.29 | 45.67 | 44.11 |
|  | 0.8 | 46.83 | 47.22 | 46.44 |
|  | 1 | 56.14 | 55.37 | 56.53 |
|  | 1.2 | 58.09 | 58.86 | 58.86 |
| F1 | 0.2 | 37.13 | 37.52 | 38.68 |
|  | 0.4 | 39.46 | 40.62 | 39.46 |
|  | 0.6 | 49.55 | 48.38 | 49.16 |
|  | 0.8 | 54.59 | 55.37 | 56.92 |
|  | 1 | 54.59 | 56.14 | 45.67 |
| F2 | 0.2 | 24.32 | 24.32 | 23.93 |
|  | 0.4 | 30.53 | 30.14 | 32.08 |
|  | 0.6 | 38.29 | 40.62 | 44.50 |
|  | 0.8 | 54.98 | 55.37 | 56.14 |
|  | 1 | 61.19 | 60.41 | 60.41 |
| F3 | 0.4 | 34.41 | 34.02 | 37.90 |
|  | 0.6 | 49.16 | 47.61 | 46.44 |
|  | 0.8 | 55.76 | 55.37 | 56.92 |
|  | 1 | 60.03 | 60.41 | 58.86 |
|  | 1.2 | 60.03 | 61.19 | 60.80 |
| F4 | 0.4 | 47.61 | 46.83 | 47.22 |
|  | 0.6 | 54.20 | 55.76 | 54.98 |
|  | 0.8 | 55.76 | 55.76 | 55.37 |
|  | 1 | 61.58 | 61.97 | 61.97 |
|  | 1.2 | 53.43 | 50.32 | 54.59 |
| GSH | 0.1 | ND | ND | ND |
|  | 0.2 | 33.9909 | 35.915 | 35.9286 |
|  | 0.4 | 47.10 | 45.18 | 46.48 |
|  | 0.6 | 52.13 | 55.62 | 51.00 |
|  | 0.8 | 64.57 | 63.92 | 60.37 |

**Data Table 3**

**Hydroxyl scavenging activity (2mg/mL protein concentration) of crude-MMPH and its ultrafiltration peptide fraction**

|  |  |  |  |
| --- | --- | --- | --- |
| Sample | Hydroxyl scavenging activity (%) | | |
|  | Rep.1 | Rep.2 | Rep.3 |
| GSH | 99.04 | 98.41 | 98.59 |
| Crude | 39.88 | 40.42 | 39.44 |
| F1 | 50.02 | 49.90 | 49.00 |
| F2 | 35.36 | 34.65 | 35.52 |
| F3 | 26.82 | 26.73 | 26.27 |
| F4 | 54.94 | 54.88 | 53.67 |

**Data Table 3**

**Superoxide scavenging activity of crude-MMPH and its ultrafiltration peptide fraction**

| Concentration (mg/mL) | Sample | Rep. | Superoxide scavenging activity (%) |
| --- | --- | --- | --- |
| 0.2 | GSH | 1 | 39.57 |
|  |  | 2 | 41.80 |
|  |  | 3 | 39.68 |
|  | Crude-MMPH | 1 | 19.63 |
|  |  | 2 | 19.63 |
|  |  | 3 | 17.76 |
|  | F1 | 1 | 19.63 |
|  |  | 2 | 19.63 |
|  |  | 3 | 17.76 |
|  | F2 | 1 | 30.84 |
|  |  | 2 | 28.97 |
|  |  | 3 | 28.97 |
|  | F3 | 1 | 30.84 |
|  |  | 2 | 30.84 |
|  |  | 3 | 27.10 |
|  | F4 | 1 | 32.71 |
|  |  | 2 | 30.84 |
|  |  | 3 | 34.58 |
| 0.4 | GSH | 1 | 45.38 |
|  |  | 2 | 48.71 |
|  |  | 3 | 49.07 |
|  | Crude-MMPH | 1 | 34.58 |
|  |  | 2 | 32.71 |
|  |  | 3 | 34.58 |
|  | F1 | 1 | 28.97 |
|  |  | 2 | 27.10 |
|  |  | 3 | 28.97 |
|  | F2 | 1 | 30.84 |
|  |  | 2 | 28.97 |
|  |  | 3 | 32.71 |
|  | F3 | 1 | 32.71 |
|  |  | 2 | 34.58 |
|  |  | 3 | 28.97 |
|  | F4 | 1 | 40.19 |
|  |  | 2 | 42.06 |
|  |  | 3 | 40.19 |

**Superoxide scavenging activity of crude-MMPH and its ultrafiltration peptide fraction**

**(Cont. 1)**

| Concentration (mg/mL) | Sample | Rep. | Superoxide scavenging activity (%) |
| --- | --- | --- | --- |
| 0.6 | GSH | 1 | 51.26 |
|  |  | 2 | 52.04 |
|  |  | 3 | 54.79 |
|  | Crude-MMPH | 1 | 38.32 |
|  |  | 2 | 34.58 |
|  |  | 3 | 34.58 |
|  | F1 | 1 | 30.84 |
|  |  | 2 | 36.45 |
|  |  | 3 | 30.84 |
|  | F2 | 1 | 36.45 |
|  |  | 2 | 38.32 |
|  |  | 3 | 38.32 |
|  | F3 | 1 | 34.58 |
|  |  | 2 | 30.84 |
|  |  | 3 | 36.45 |
|  | F4 | 1 | 43.93 |
|  |  | 2 | 45.79 |
|  |  | 3 | 47.66 |
| 0.8 | GSH | 1 | 63.04 |
|  |  | 2 | 64.23 |
|  |  | 3 | 61.91 |
|  | Crude-MMPH | 1 | 40.19 |
|  |  | 2 | 42.06 |
|  |  | 3 | 38.32 |
|  | F1 | 1 | 38.32 |
|  |  | 2 | 34.58 |
|  |  | 3 | 38.32 |
|  | F2 | 1 | 40.19 |
|  |  | 2 | 38.32 |
|  |  | 3 | 40.19 |
|  | F3 | 1 | 40.19 |
|  |  | 2 | 43.93 |
|  |  | 3 | 40.19 |
|  | F4 | 1 | 49.53 |
|  |  | 2 | 45.79 |
|  |  | 3 | 47.66 |

**Superoxide scavenging activity of crude-MMPH and its ultrafiltration peptide fraction**

**(Cont. 2)**

| Concentration (mg/mL) | Sample | Rep. | Superoxide scavenging activity (%) |
| --- | --- | --- | --- |
| 1.0 | GSH | 1 | 82.60 |
|  |  | 2 | 79.69 |
|  |  | 3 | 78.58 |
|  | Crude-MMPH | 1 | 51.40 |
|  |  | 2 | 53.27 |
|  |  | 3 | 53.27 |
|  | F1 | 1 | 42.06 |
|  |  | 2 | 47.66 |
|  |  | 3 | 42.06 |
|  | F2 | 1 | 43.93 |
|  |  | 2 | 43.93 |
|  |  | 3 | 42.06 |
|  | F3 | 1 | 40.19 |
|  |  | 2 | 38.32 |
|  |  | 3 | 42.06 |
|  | F4 | 1 | 66.36 |
|  |  | 2 | 64.49 |
|  |  | 3 | 64.49 |

**Data Table 4**

**Ferric reducing antioxidant power (FRAP) of crude-MMPH and its ultrafiltration peptide fraction**

| Sample | Concentration (mg/mL) | Rep.1 | Rep.2 | Rep.3 |  |  |
| --- | --- | --- | --- | --- | --- | --- |
| GSH | 1 | 0.64 | 0.66 | 0.65 |  |  |
|  | 0.8 | 0.52 | 0.55 | 0.53 |  |  |
|  | 0.6 | 0.43 | 0.45 | 0.41 |  |  |
|  | 0.4 | 0.28 | 0.28 | 0.29 |  |  |
|  | 0.2 | 0.20 | 0.20 | 0.20 |  |  |
| Crude-MMPH | 1 | 0.20 | 0.21 | 0.21 |  |  |
|  | 0.8 | 0.16 | 0.17 | 0.16 |  |  |
|  | 0.6 | 0.12 | 0.13 | 0.12 |  |  |
|  | 0.4 | 0.07 | 0.07 | 0.07 |  |  |
|  | 0.2 | 0.04 | 0.04 | 0.04 |  |  |
| F1 | 1 | 0.23 | 0.24 | 0.26 |  |  |
|  | 0.8 | 0.23 | 0.23 | 0.23 |  |  |
|  | 0.6 | 0.12 | 0.12 | 0.12 |  |  |
|  | 0.4 | 0.11 | 0.11 | 0.11 |  |  |
|  | 0.2 | 0.06 | 0.06 | 0.06 |  |  |
| F2 | 1 | 0.21 | 0.22 | 0.20 |  |  |
|  | 0.8 | 0.18 | 0.18 | 0.18 |  |  |
|  | 0.6 | 0.13 | 0.14 | 0.14 |  |  |
|  | 0.4 | 0.08 | 0.08 | 0.08 |  |  |
|  | 0.2 | 0.04 | 0.04 | 0.04 |  |  |
| F3 | 1 | 0.15 | 0.17 | 0.15 |  |  |
|  | 0.8 | 0.13 | 0.14 | 0.13 |  |  |
|  | 0.6 | 0.10 | 0.11 | 0.11 |  |  |
|  | 0.4 | 0.07 | 0.06 | 0.06 |  |  |
|  | 0.2 | 0.01 | 0.02 | 0.02 |  |  |
| F4 | 1 | 0.10 | 0.10 | 0.10 |  |  |
|  | 0.8 | 0.07 | 0.07 | 0.07 |  |  |
|  | 0.6 | 0.06 | 0.06 | 0.05 |  |  |
|  | 0.4 | 0.03 | 0.03 | 0.03 |  |  |
|  | 0.2 | 0.00 | 0.00 | 0.00 |  |  |

**Data Table 5**

**Metal chelation activity of crude-MMPH and its ultrafiltration peptide fraction**

| Sample | Concentration (mg/mL) | Rep.1 | Rep.2 | Rep.3 |  |
| --- | --- | --- | --- | --- | --- |
| GSH | 0.6 | 16.61982 | 14.11541 | 16.09946 |  |
|  | 1 | 36.30288 | 37.2266 | 37.83182 |  |
|  | 3 | 50.00009 | 49.56383 | 50.25136 |  |
|  | 5 | 53.03552 | 51.52966 | 51.59398 |  |
| Crude-MMPH | 0.6 | 9.94575 | 8.318264 | 8.137432 |  |
|  | 1 | 11.03074 | 11.57324 | 11.39241 |  |
|  | 3 | 22.06148 | 21.69982 | 20.79566 |  |
|  | 5 | 41.41049 | 41.04882 | 40.3255 |  |
| F1 | 0.6 | 6.690778 | 7.052441 | 5.244123 |  |
|  | 1 | 8.499096 | 6.148282 | 6.148282 |  |
|  | 3 | 21.33816 | 22.60398 | 21.15732 |  |
|  | 5 | 39.783 | 38.87884 | 37.25136 |  |
| F2 | 0.6 | 9.222423 | 8.860759 | 8.137432 |  |
|  | 1 | 9.403255 | 6.871609 | 7.9566 |  |
|  | 3 | 23.68897 | 26.94394 | 25.31646 |  |
|  | 5 | 38.15552 | 37.43219 | 35.62387 |  |
| F3 | 0.6 | 7.052441 | 5.786618 | 6.509946 |  |
|  | 1 | 8.499096 | 9.041591 | 8.137432 |  |
|  | 3 | 38.15552 | 39.96383 | 40.14467 |  |
|  | 5 | 41.22966 | 40.86799 | 41.41049 |  |
| F4 | 0.6 | 8.860759 | 8.318264 | 9.041591 |  |
|  | 1 | 11.9349 | 13.38156 | 13.38156 |  |
|  | 3 | 40.86799 | 43.7613 | 42.49548 |  |
|  | 5 | 43.94213 | 43.58047 | 44.3038 |  |
|  |  |  |  |  |  |

**Data Table 6**

**Amino acid composition of crude-MMPH and its ultrafiltration peptide fractions**

| **Group** | **Amino acid** | **Crude-MMPH Content (mg / g Protein)** | | | | | | **Total amino acid  (% w/w Protein)** | | | | | | | |
| --- | --- | --- | --- | --- | --- | --- | --- | --- | --- | --- | --- | --- | --- | --- | --- |
|  |  | **Total amino acids** | | **Free amino acids** | | **amino acid in peptide form** | | **F1** | | **F2** | | **F3** | | **F4** | |
|  |  | **Rep.1** | **Rep.2** | **Rep.1** | **Rep.2** | **Rep.1** | **Rep.2** | **Rep.1** | **Rep.2** | **Rep.1** | **Rep.2** | **Rep.1** | **Rep.2** | **Rep.1** | **Rep.2** |
| **Acidic**  **(- charge)** | **Asp** | 107.17 | 101.61 | 1.09 | 1.03 | 106.08 | 100.58 | 14.33 | 14.12 | 14.93 | 12.75 | 13.34 | 14.14 | 11.20 | 12.33 |
|  | **Glu** | 161.22 | 164.10 | 17.28 | 16.56 | 143.94 | 147.54 | 18.31 | 18.28 | 18.11 | 17.78 | 18.90 | 17.56 | 19.39 | 18.05 |
| **Basic**  **(+ charge)** | **Lys** | 67.78 | 76.44 | 28.80 | 29.08 | 38.98 | 47.36 | 8.28 | 8.01 | 8.62 | 8.01 | 7.54 | 8.45 | 6.57 | 7.21 |
|  | **Arg** | 66.02 | 67.98 | 21.91 | 22.29 | 44.11 | 45.69 | 6.23 | 7.03 | 6.32 | 7.10 | 6.53 | 6.90 | 7.18 | 6.52 |
|  | **His** | 19.85 | 20.92 | 11.16 | 12.00 | 8.69 | 8.92 | 3.08 | 4.01 | 2.58 | 3.56 | 2.36 | 3.14 | 2.62 | 3.41 |
| **Hydropho-bic** | **Gly** | 38.56 | 35.00 | 4.88 | 6.10 | 33.68 | 28.90 | 3.50 | 2.87 | 3.44 | 4.12 | 4.49 | 2.97 | 3.84 | 4.12 |
|  | **Ala** | 39.13 | 40.91 | 10.20 | 10.52 | 28.93 | 30.39 | 3.92 | 4.32 | 4.69 | 3.24 | 4.47 | 3.34 | 5.28 | 4.47 |
|  | **Val** | 35.42 | 37.54 | 6.86 | 6.50 | 28.56 | 31.04 | 4.86 | 4.10 | 5.49 | 4.86 | 4.98 | 5.62 | 4.35 | 5.69 |
|  | **ILeu** | 35.56 | 38.16 | 3.75 | 3.51 | 31.81 | 34.65 | 4.33 | 5.11 | 4.15 | 5.12 | 6.13 | 3.21 | 2.88 | 4.23 |
|  | **Leu** | 62.82 | 63.03 | 22.19 | 22.39 | 40.63 | 40.64 | 6.28 | 6.67 | 7.27 | 5.99 | 7.22 | 6.17 | 9.12 | 8.08 |
|  | **Pro** | 47.24 | 48.78 | 3.50 | 3.64 | 43.74 | 45.14 | 4.81 | 5.12 | 4.75 | 6.43 | 6.35 | 5.98 | 4.12 | 3.11 |
|  | **Met** | 7.92 | 9.88 | 4.68 | 4.52 | 3.24 | 5.36 | 1.14 | 0.85 | 1.39 | 0.87 | 0.74 | 1.43 | 1.22 | 1.98 |
|  | **Cys** | 3.73 | 4.21 | 0.35 | 0.03 | 3.38 | 4.18 | 2.39 | 2.14 | 0.37 | 1.28 | 0.22 | 0.98 | 0.18 | 0.41 |
| **Aromatic** | **Tyr** | 27.10 | 30.95 | 4.43 | 6.05 | 22.67 | 24.90 | 3.67 | 3.78 | 3.80 | 2.93 | 3.03 | 3.84 | 3.40 | 4.21 |
|  | **Phe** | 44.63 | 46.59 | 7.17 | 6.95 | 37.46 | 39.64 | 4.89 | 4.56 | 5.38 | 4.77 | 3.91 | 6.43 | 7.23 | 5.92 |
|  | **Trp** | 5.83 | 6.27 | 1.30 | 1.06 | 4.53 | 5.21 | 0.72 | 0.67 | 0.46 | 1.02 | 0.24 | 1.32 | 0.33 | 1.32 |
| **Hydrophilic** | **Ser** | 52.59 | 55.95 | 14.32 | 13.08 | 38.27 | 42.87 | 5.68 | 5.27 | 05.03 | 6.48 | 6.23 | 5.11 | 7.03 | 5.96 |
|  | **Thr** | 32.20 | 34.62 | 5.74 | 6.12 | 26.46 | 28.50 | 3.58 | 3.09 | 3.22 | 3.69 | 3.32 | 3.41 | 4.06 | 2.98 |
| **Total** | | 854.77 | 882.94 | 169.61 | 171.43 | 685.16 | 711.51 | 100.00 | 100.00 | 100.00 | 100.00 | 100.00 | 100.00 | 100.00 | 100.00 |
